# The importance of gravity vector on adult mammalian organisms: Effects of hypergravity on mouse testis

**DOI:** 10.1371/journal.pone.0282625

**Published:** 2023-09-29

**Authors:** Valentina Bonetto, Valeria Magnelli, Maurizio Sabbatini, Flavia Caprì, Jack J. W. A. van Loon, Sara Tavella, Maria Angela Masini

**Affiliations:** 1 Department of Science and Technology Innovation, University of Eastern Piedmont (UPO), Alessandria (AL), Italy; 2 Department of Oral and Maxillofacial Surgery/Pathology, Amsterdam Movement Sciences & Amsterdam Bone Center (ABC), Amsterdam UMC Location Vrije Universiteit Amsterdam & Academic Center for Dentistry Amsterdam (ACTA), Amsterdam, The Netherlands; 3 Life Support and Physical Sciences Section (TEC-MMG), European Space Agency (ESA), European Space Research and Technology Centre (ESTEC), Noordwijk, The Netherland; 4 Department of Experimental Medicine, University of Genoa, Genoa (GE), Italy; National Institute of Child Health and Human Development (NICHD), NIH, UNITED STATES

## Abstract

In the age of space exploration, the effect of hypergravity on human physiology is a relatively neglected topic. However, astronauts have several experiences of hypergravity during their missions. The main disturbance of altered gravity can be imputed to cell cytoskeleton alteration and physiologic homeostasis of the body. Testis has proved to be a particularly sensible organ, subject to environmental alteration and physiological disturbance. This makes testis an organ eligible for investigating the alteration following exposure to altered gravity. In our study, mice were exposed to hypergravity (3*g* for 14 days) in the Large Diameter Centrifuge machine (ESA, Netherland). We have observed a morphological alteration of the regular architecture of the seminiferous tubules of testis as well as an altered expression of factors involved in the junctional complexes of Sertoli cells, responsible for ensuring the morpho-functional integrity of the organ. The expression of key receptors in physiological performance, such as Androgen Receptors and Interstitial Cells Stimulating Hormone receptors, was found lower expressed. All these findings indicate the occurrence of altered physiological organ performance such as the reduction of the spermatozoa number and altered endocrine parameters following hypergravity exposure.

## Introduction

Space exploration represents the upcoming frontier of human being, in particular as concerning the incoming project on Mars, which means a long stay in the space environment with prolonged gravitational alterations [1]. In this scenario, the influence of exposure to a gravitational environment other than the Earth’s for upcoming time is an important topic to consider due to its influence on the human physiology.

The gravitational force is an essential physical component of the Earth’s environment, and gravity is known to exert a strong influence at the cellular level. Experimental investigations have found that cell cytoskeleton is mainly affected [2], and changes in intracellular molecular pathways were also observed [3].

In the last years the cellular and physiological effects of microgravity have gained increased attention, following the concerns about a progressive increase in the amount of time astronauts spend at low gravity [1, 4–6]. But there have been rather few studies about hypergravity [7, 8].

Hypergravity, which varies between 2-4*g*, is experienced for a very short time compared to microgravity environment during the entire space mission. Probably due to the last reason, in recent years there has been a tendency to minimize the reported consequences, as can be assessed from the scarce scientific papers available.

Nevertheless, astronauts have several experiences of hypergravity during their mission, such as during the exit from terrestrial orbit or during a return to earth ground, where the possible use of space-shuttle, can impose phases of prolonged high-gravity flight [9].

The hypergravity condition mainly affects hormonal balance and homeostasis regulation together with the body fluid compartment disturbances and, even although the details of the hormone dysregulation are not known, hypergravity clearly induces multiple changes in the interrelationship among hormones and alterations in the sensitivity of responding systems [10].

Casey et al. [11] reported a complex change in circadian rhythm which caused a decreased rate of mammary metabolic activity and increased pup mortality in rats, following exposure to hypergravity. The consequences of hypergravity have also been proved on calcitonin released by thyroid parafollicular cells together with significant reduction in the thickness of cortical bone [12]. Furthermore, spaceflight has been shown to significantly affect the reproductive physiology of the testis [1].

Testis, the male organ of the reproductive system, is a glandular organ, that for its correct functionality as gametogenic reproductive organ, is widely dependent on correct relationship with several endocrine factors. Following this particularly dependence on the complex endocrine environment of the body, testis is considered a good candidate to study in general the occurrence of body homeostasis alteration [13].

One of the main hitches in studying the biological responses to hypergravity has been in the past the lack of experimental devices to reproduce the hypergravity environment in terrestrial laboratory and therefore to carry out experimental research on animal model for long time period. Previously, the only solution was represented by an agreement with space missions on International Space Station (ISS), where experiment on living animals were performed by properly trained astronauts.

Now the hypergravity experiments can be performed quite easily on live animals by the use of the Large Diameter Centrifuge machine. Centrifugation is, in fact, a good ground-based model to simulate altered gravity which occurs during space missions [14]. The Large Diameter Centrifuge machine, of European Spatial Agency (ESA), placed in the Netherland, is built with peripheral cages coupled by an arm to a central rotor. The cages are equipped with control device for monitoring animal condition. This equipment allows to study different experimental animal models. Further the animal samples can be tracked under hypergravity condition for a long time.

In the present work we have investigated the effect of hypergravity on the morphological structure and endocrine regulation of the testis in mice, hold in hypergravity environment for several days. Furthermore, the study is also presented to show the reliability and reproducibility of the data using the Large Diameter Centrifuge machine.

## Materials and methods

### Animal care

In all phases of the hypergravity experiment animals were handled according to internationally accepted principles for care of laboratory animals (E.E.C. Council Directive 86/609, OJL358,1, Dec. 12, 1987).

Formal approval to conduct the described experiments was obtained from the Public Veterinary Health Department of the Italian Ministry of Health (prot. n. 4347-09/03/2009-DGSA.P.) and the University of Genoa (Genoa, Italy), where the naïve group mice were stabularized.

The authors of this article were not directly involved in executing the animal maintenance part of the experiments. Instead, they were responsible for the mice management at the end of the hypergravity experiment and were involved in the specific tissue collection and analysis.

### Experimental design

Experiment in hypergravity was performed at the Large Diameter Centrifuge, located at European Space Research and Technology Centre (ESTED, Noordwijk, NL). This instrument is composed by several peripheral cages coupled by an arm to a central rotor. A cage placed up the body of central rotor is used as control. The rotor activity induces a rotational movement to the peripheral cages responsible of a centrifugal force mimicking the increased gravity. Each cage is equipped with camera and control device for observing animal behaviour, maintaining standard environmental conditions and monitoring vital parameters [15].

In this experiment 6 mice (C57BL/10J, 8 weeks-old), individually housed in the peripheral cages, were used, imposed to a 3*g* of hypergravity for 14 days. Six mice were placed for the same time in the central cages of the rotor, where hypergravity was not imposed. They were used as control (sham)-group of hypergravity experiment. A naïve control group was made in institutional stabularium of University of Genoa, where five mice (C57BL/10J,8 weeks-old) were housed in standard stabularium cages for 14 days.

At the end of the experimental period, mice were euthanised by anaesthesia under 0.5–2% isoflurane gas (O_2_: 95.0%), sacrificed by decollation.

Testes, including epididymis, were removed bilaterally. The right testes were quickly frozen in liquid nitrogen and stored in dry ice. The left testes were divided in two halves: one was fixed in 4% paraformaldehyde and stored in phosphate-buffered saline (PBS 1X), the other was quickly frozen in liquid nitrogen and stored in dry ice. Specimens were then shipped to dep. of Science and Technological Innovation (DISIT) of University of Eastern Piedmont (UPO), where the investigative protocols were performed, and data analysed.

The fixed samples were processed for embedding in paraffin wax. Consecutive serial sections were cut (5 μm thick) and mounted on silanized-slides. Then slides were dewaxed, rehydrated through descending series of ethanol and alternate slides were submitted to investigative protocols as detailed below. The frozen samples were used for PCR protocol.

The histological microarchitecture of the organ was investigated by Masson’s trichromic staining.

### Immunohistochemistry and immunofluorescence

Three slides for each sample were decorated with each primary antibodies detailed below.

Immunohistochemical protocol was applied to investigate the distribution of 3β-steroid dehydrogenase (3β-HSD) and 17β-steroid dehydrogenase (17β-HSD). Briefly the dewaxed and hydrated slides were incubated overnight at 4°C with primary antibody against 3β-HSD (raised in mouse; Santa Cruz Biotechnology Inc.; dilution of 1:50); and 17β-HSD (raised in mouse; Santa Cruz Biotechnology Inc.; dilution of 1:50); after washing in PBS respectively anti-mouse secondary antibody, mouse IgGκ BP-HRP (Santa Cruz Biotechnology Inc.; dilution of 1:25) was added to reveal specific site by 3,3-diaminobenzidine (DAB) colorimetric reaction.

Immunofluorescence protocol was applied to investigate the distribution of adhesion protein. Briefly slides were incubated overnight at 4°C with primary antibody against Claudin-1 (monoclonal antibody raised in mouse, Alexa Fluor 488, Invitrogen; dilution of 1:100), Claudin-4 (monoclonal antibody raised in mouse, Alexa Fluor 594, Invitrogen; dilution of 1:100), Occludin (monoclonal antibody raised in mouse, Alexa Fluor 594, Invitrogen; dilution of 1:100), Connexin-43 (polyclonal antibody raised in rabbit, Invitrogen; dilution of 1:200) and the Sex Hormone Binding Globulin (SHBG; polyclonal antibody raised in rabbit, Santa Cruz Biotechnology Inc.; dilution of 1:50). After washing in PBS 1X, a second layer of fluoresceine-isothiocyanate conjugated gamma-globulins, goat anti-mouse (dilution of 1:100; Sigma-Aldrich) or goat anti-rabbit (dilution of 1:100; Sigma-Aldrich) following the source of the primary antisera was applied for 30 min in moist chamber at room temperature. Sections were rinsed in PBS 1X, mounted with glycerol-PBS 1X (1:9) and examined under a Leica epifluorescence microscope.

The specificity of the immunostaining (both colorimetric and fluorescent) was verified by omitting the primary antibody or by replacing the primary antiserum with nonimmune, in both cases no immunostaining was detected.

### RT-qPCR

RNA from frozen samples was isolated by the acid phenol-chloroform procedure using the Trizol reagent (Sigma). Quality of isolated RNA was checked by electrophoresis on 1.5% agarose gel. Concentrations and purities of the isolated RNA were assessed by absorption spectroscopy. Aliquots of 1.5 mg RNA were reverse-transcribed into cDNA using 200 units RevertAid H Minus M-MuLV Reverse Transcriptase (Fermentas, MMedical, Milan, Italy), in presence of 200 pmol of poly-T18mer (TIB Mol Biol, Italia), 1 mM dNTPs (Fermentas) at 42°C for 60 min in a reaction volume of 20 ml. The cDNA was used to amplify the genes of interest using a Chromo 4TM System real-time PCR apparatus (Biorad Italy, Milan). Proper aliquots of the RT mixture were diluted to a final volume of 20 ml in presence of iTaq SYBR Green Supermix with Rox (Biorad) and 0.25 mM of each specific primer pairs (TibMolBiol, Genoa, Italy). The primer pairs used are shown in Table 1. Thermal protocol consisted of 3-min initial denaturation at 95°C followed by 40 cycles: 5 s at 95°C and 20 s at 60°C. A melting curve of PCR products (55–94°C) was also performed to rule out the presence of artifacts. Relative quantification of each gene expression was calculated according to comparative Ct method using the Biorad software tool Genex-Gene Expression MacroTM.

**Table 1:** Primer sequences used in RT-qPCR analysis.

**AR**	*Forward*	5’ — GCAGCTTGTGCATGTGGTCA
	*Reverse*	5’ — AATACCATCAGTCCCATCCAGGAA
**ICSHR**	*Forward*	5’ — CAGGAATTTGCCGAAGAAAGAACAGAATT
	*Reverse*	5’ — CAGAAGTCATAATCGTAATCCCAGCCA
**FSHR**	*Forward*	5’ — CCTCTGCCAAGATAGCAAGGTA
	*Reverse*	5’ — CTCCAGGTCCCCAAATCCAGA
**IL-1β**	*Forward*	5’ — CAGGCAGGCAGTATCACTCA
	*Reverse*	5’ — GGTGCTCATGTCCTCATCCT

Expression of the genes of interest was normalized using the expression levels of GAPDH as housekeeping gene and the normalized expression was then expressed as relative quantity of mRNA (relative expression) with respect to laboratory and ground control samples.

Statistical analysis was performed using Kruskal-Wallis non-parametric test followed by post-hoc Dunn test by statistical software PRISM 2.01 (GraphPad Software Inc., CA, USA). p values < 0.05 were considered significant.

### Tunel

Apoptosis was detected using an in situ TdT technique (In situ Cell Death Detenction Kit, fluorescein; Roche, Applied-Science). Three sections for each sample were incubated with permeabilization solution (0,1% Triton X-100, 0,1% sodium citrate) freshly prepared for 10 minutes at room temperature, washed with PBS 1X (pH 7.2–7.4) and incubated at 37°C for one hour with the TUNEL reaction mixture. At the end of incubation slides were washed twice in PBS 1x, mounted with PBS 1x and analyzed under fluorescence microscope (excitation wavelength 488 nm and detection 515–565 nm).

## Results

One out of six mice exposed to hypergravity deceased. The other five mice exposed to hypergravity environment resulted alive at the end of experiments; they presented weight loss (about 15%), probably due to greater energy expenditure during hypergravity period. No evident suffering status was observed. No weight loss or suffering status was observed in mice used as control.

Morphological, immunohistochemical and molecular analysis performed on control and naïve group rats showed similar evidence and results. No more concerns were detailed about comparison between control and naïve group rats.

### Histomorphology

**Fig 1:**
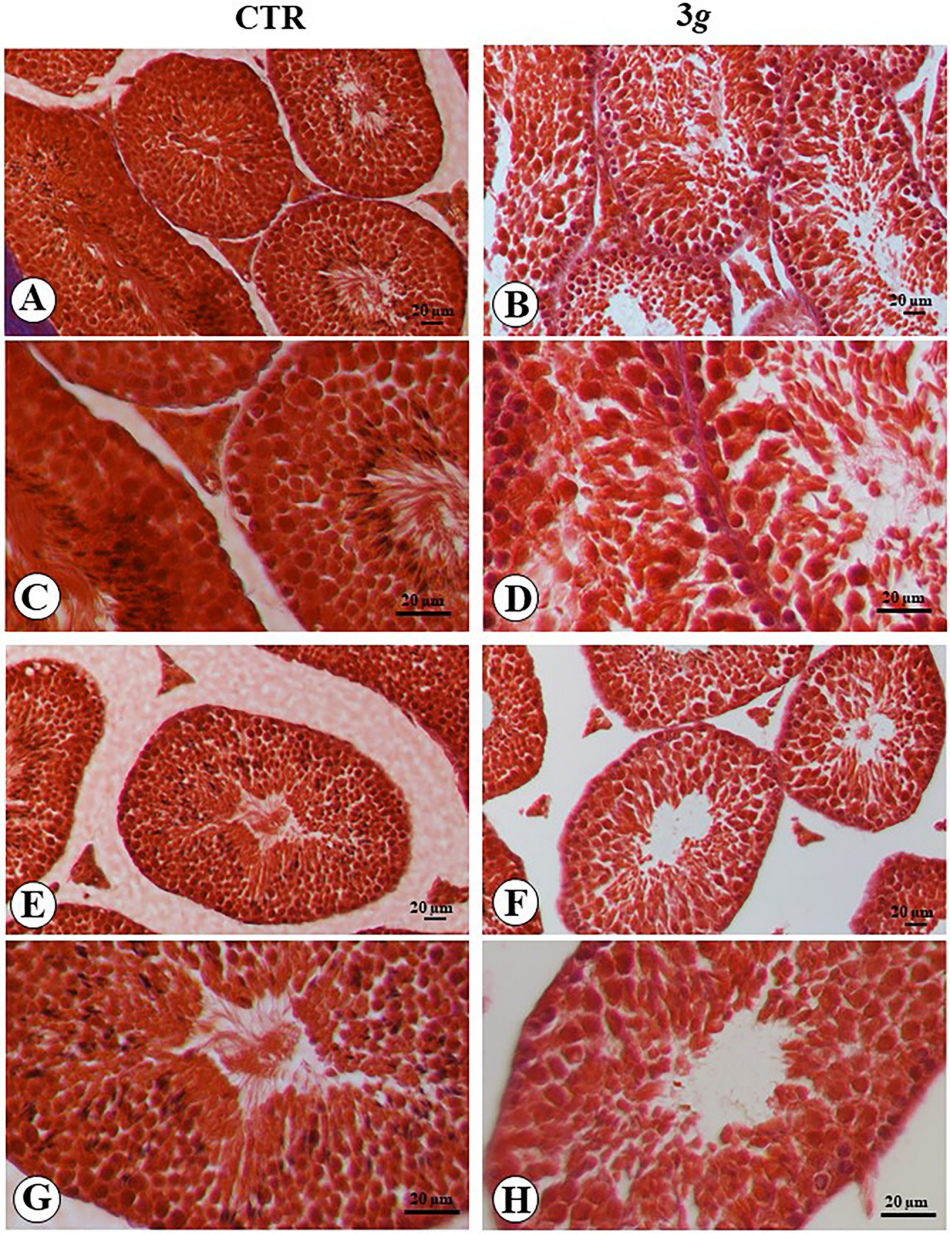
Microphotograph panel illustrating the microanatomy of testis seminiferous tubules in normogravity (A,C,E,G) and following 3*g* exposure (B,D,F,H). The tubules presented are detailed in the bottom part (A-D) and the upper part (E-H) of testis seminiferous tubules. Note the irregular disposition of spermatogonia cells and Sertoli cells, and the reduction of tubules thickness following 3 *g* exposure. CTR: normogravity; 3 *g*: hypergravity.

The normal microanatomy of testis in the bottom part of each testicular lodge showed elongated and convoluted seminiferous tubules profiles. A limited interstitial tissue is present between outline of the tubules, where cluster of endocrine Leydig cells can be observed (Fig 1A and 1C). In the upper part of the testicular lodge, seminiferous tubules display a lesser convoluted and a more roundish profile with evident interstitial tissue between tubules outline, where clusters of endocrine Leydig cells can be observed (Fig 1E, 1G). In both part of seminiferous tubules, the stratified epithelium (forming the wall of the seminiferous tubules) clearly showed the composition of two main cell types: spermatogenic cells and Sertoli cells (Fig 1C, 1G). Spermatogenic cells appeared regularly roundish and stacked along the epithelium thickness. The germinal layer of epithelium was composed of cells aligned in monolayer located immediately above the basal lamina, it was formed by roundish cells showing a hypochromatic nucleus (spermatogonia cells) (Fig 1C, 1G). Spermatogenic cells appeared as large round cells with large nuclei and rounded outlines. The last layer of the epithelium was represented by small cells, with a little and picnotic nucleus, close to the lumen, where tails of maturating spermatozoa were clearly visible (spermatid cells). Sertoli cells were visible between the spermatogenic cells, showing a large, round and well chromatic nucleus located mainly above the germinal line of the epithelium (Fig 1C, 1G).

Following exposure of mice to hypergravity (3*g*-mice), testicular morphology showed several changes. The seminiferous tubules in the bottom part of testicular lodge appear more convoluted than in the control mice, and the interstitial tissue is extremely reduced, the endocrine Leydig cells clusters appeared reduced in size (Fig 1B, 1D). In the upper part tubules appear roundish and smaller, here more interstitial tissue can be observed, where small Leydig cells clusters can be noted (Fig 1F, 1H). The germinal layer of epithelium showed small cells with hypercromatic nucleus. A reduction in the thickness of epithelium of seminiferous tubules can be observed.

Spermatocytes showed separation from each other and disorganization of the stratification consisting of small cells adjacent to large cells. A reduced number of tails of maturating spermatozoa were visible (Fig 1D, 1H). No changes were observed in rete testis tubules and epididymis tubules.

### Immunohistochemistry and immunofluorescence

Claudin-1, -4, and occludin are structural protein belonging to cell-cell tight junction. In seminiferous tubules these proteins are localized in Sertoli cells, where they contribute to maintain the haemato-testis barrier and associative coherence of these cells arranged along all tubular thickness.

**Fig 2:**
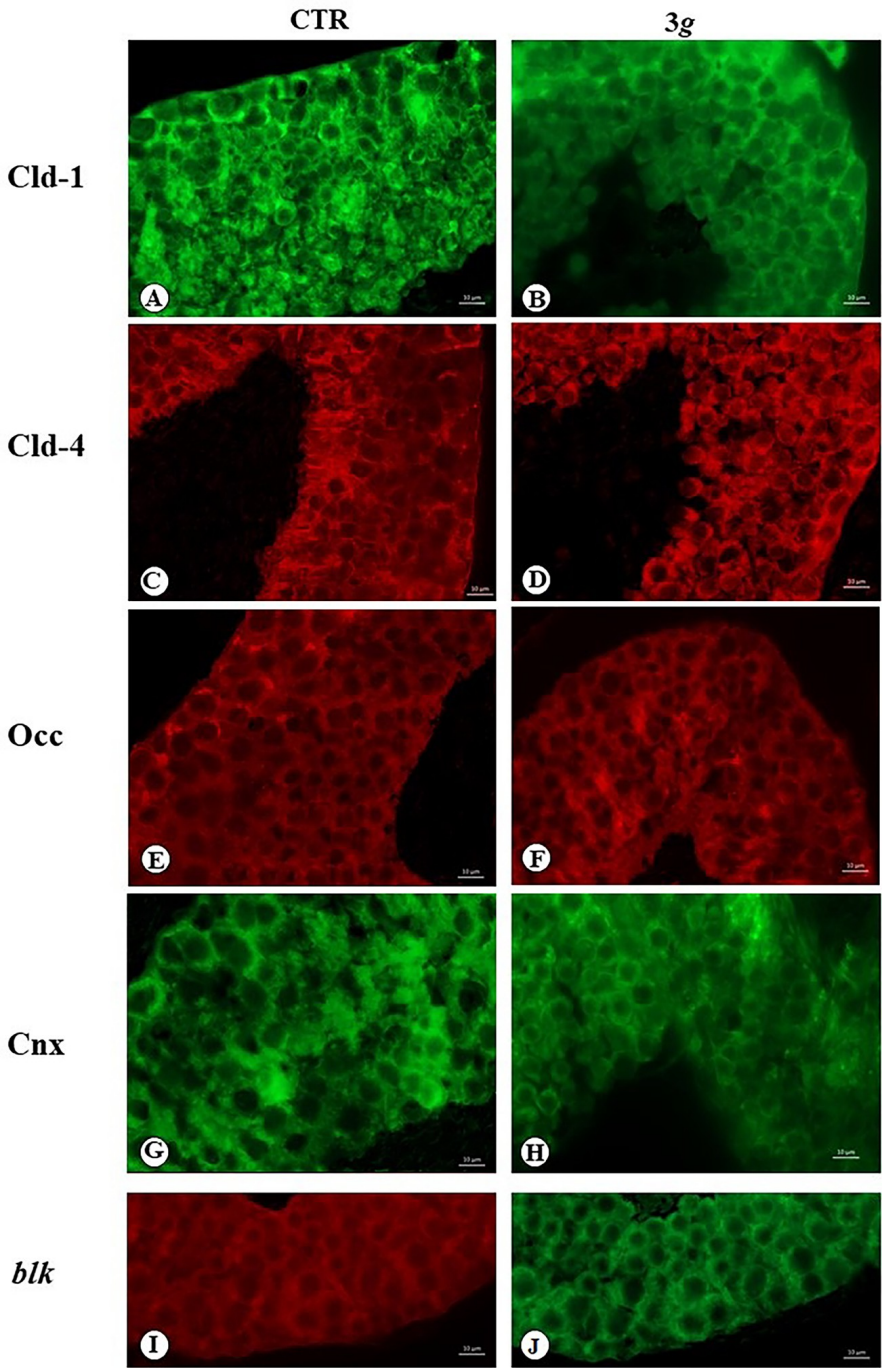
Microphotograph panel illustrating the immunofluorescence signals of cell-cell junctional protein complex occurring in the testis seminiferous tubules. A,B: Claudin-1 (Cld-1); C,D: Claudin-4 (Cld-4); E,F: Occludin (Occ); G,H: Connexin (Cnx). I,J; control immunofluorescence images, Blank (blk). Note that the analysed proteins are localized along the Sertoli cell profile. The localization pattern of analysed proteins changes following 3 *g* exposure. CTR: normogravity; 3 *g*: hypergravity.

The claudin-1 immuno-fluorescence in control mice was observed at the base and along the entire tubular thickness (Fig 2A), indicating its localization along the cellular body of Sertoli cells. Instead in 3*g*-mice, the claudin-1 signals have been observed only at the base of the tubules (Fig 2B).

The claudin-4 immuno-fluorescence in control mice was observed localized at the base and apex of seminiferous tubules (Fig 2C), while in the 3*g*-mice the fluorescence was observed arranged throughout tubules thickness (Fig 2D).

Occludin immune-fluorescence in normal mice was observed localized in the basal layer of seminiferous tubules (Fig 2E), while in 3*g*-mice immune-fluorescence was evident in the mid-zone of tubules (Fig 2F).

Connexin is a protein that mediate the specific cell-cell contact characterising the gap junction. In seminiferous tubules connexin realizes the molecular connection that supports the functional syncytium of Sertoli cells. In normal mice connexin is detected along the thickness of tubules, because it diffuses extensively along the cell body of Sertoli cells (Fig 2G). In 3*g*-mice the expression of connexin is reduced in the basal part of tubules, indicating a probable functional disconnection of apical part of Sertoli cells (Fig 2H).

**Fig 3:**
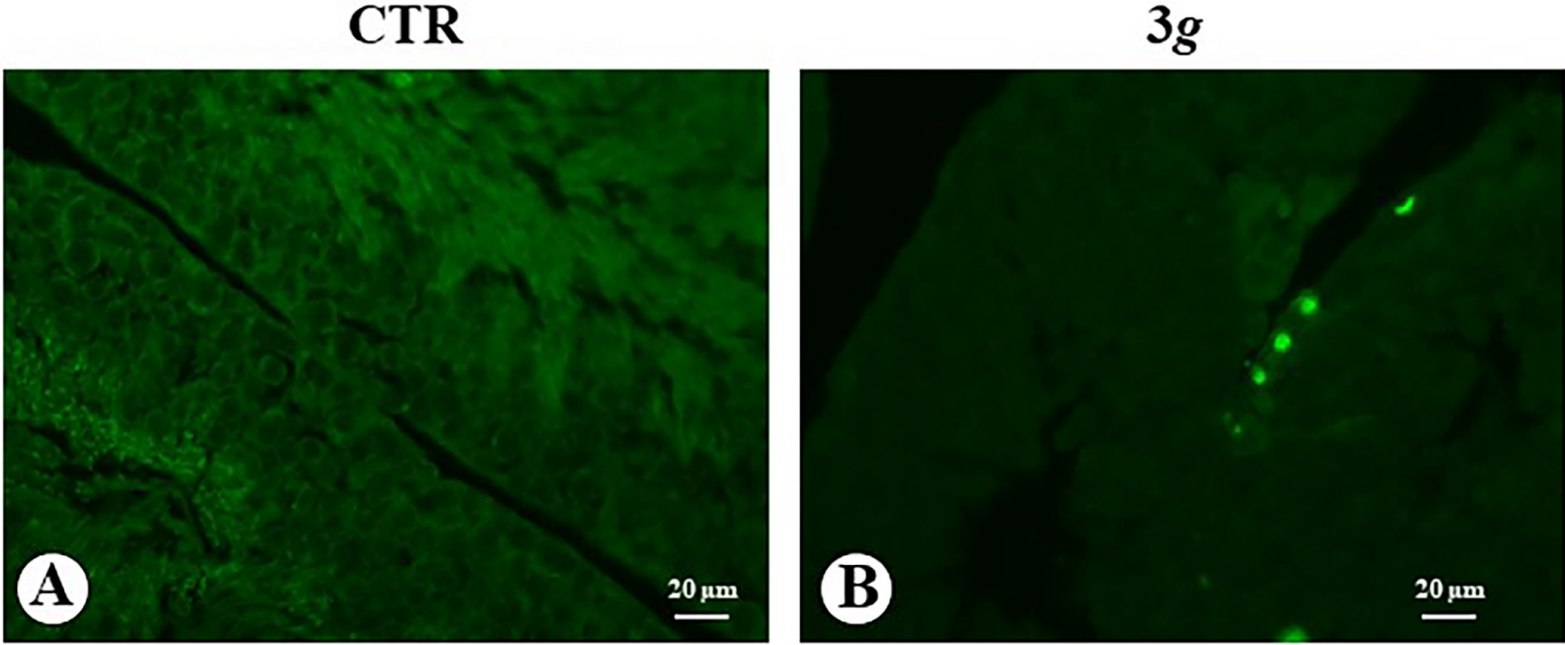
Microphotograph panel illustrating immunofluorescence TUNEL signals occurring in the testis seminiferous tubules. Note that positive signals are selectively localized on basal and well evident round nuclei, compatible with the localization of Sertoli cell nuclei. CTR: normogravity; 3 *g*: hypergravity.

The detection of apoptotic cells in seminiferous tubules of control mice by TUNEL technique showed no evidence (Fig 3A), instead in 3*g*-mice several TUNEL signal were detected along the basal portion of the tubules, where the large dimension of TUNEL-positive nuclei indicates the occurrence of apoptosis in the Sertoli cells rather than in spermatogonia cells (Fig 3B).

**Fig 4:**
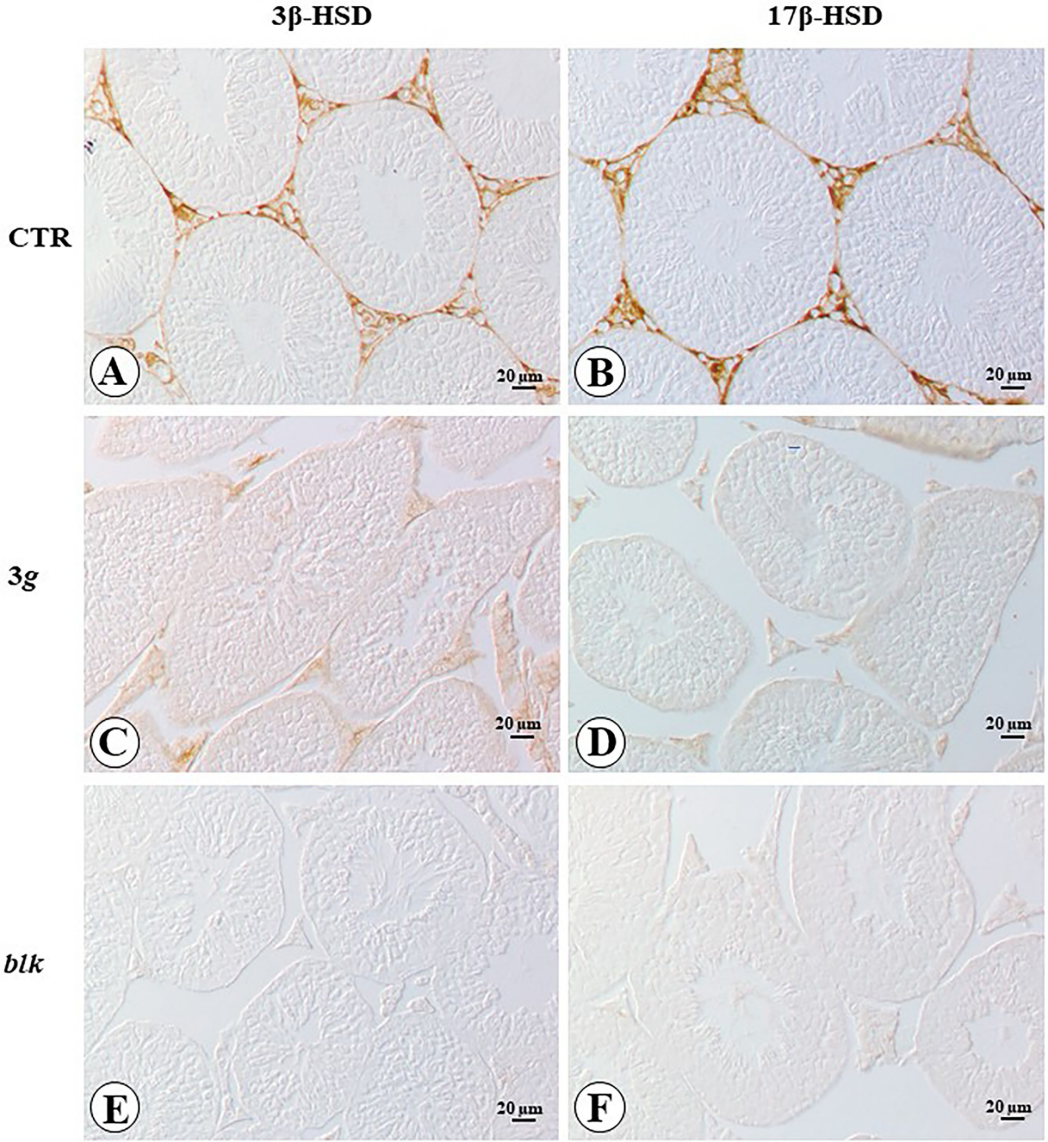
Microphotograph panel illustrating the immunohistochemistry localization and expression of 3β-HSD (A,B) and 17β-HSD (C,D), in testis of mice exposed to normogravity (A,C) or Hypergravity (B,D). E,F: control immunohistochemistry pictures, Blank (blk). Note the specific localization of the factor in the endocrine Leydig cells of the organ and the great decrease of immunopositive reaction in mice exposed to hypergravity. CTR: normogravity; 3 *g*: hypergravity.

Specific immunohistochemistry for 3β-HSD and 17β-HSD showed in either case exclusive localization in Leydig cells and in control group mice (Fig 4A and 4B). In 3*g*-mice the 3β-HSD immunohistochemistry appeared greatly reduced, whereas the 17β-HSD immunohistochemistry was completely absent (Fig 4C and 4D). Negative immunohistochemistry control slides resulted completely blank (Fig 4E and 4F).

**Fig 5:**
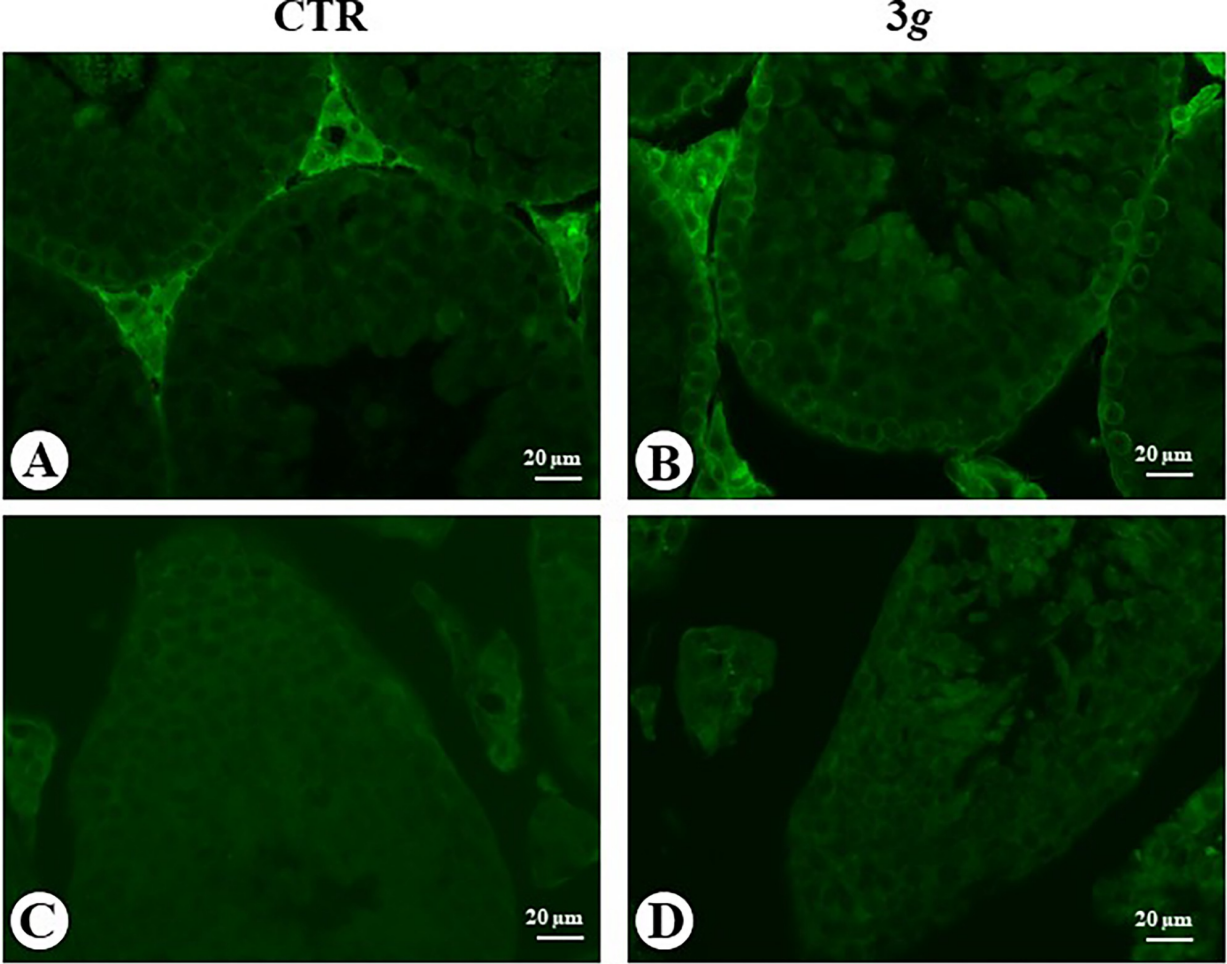
Microphotograph panel illustrating the immunofluorescence localization and expression of testosterone binding protein (SHBG) in mice testis following normogravity (A) or hypergravity (B). C,D: control immunofluorescence images, Blank. Note the extended localization of immune SHBG on basal profile of Sertoli cells following hypergravity. CTR: normogravity; 3 *g*: hypergravity.

SHBG is a testosterone binding protein whose function is to reduce the availability of circulating and active free testosterone; it is present in the basal portion of Sertoli cells. The immunofluorescence for SHBG was faintly visible along the basal profile of Sertoli cells in normal mice (Fig 5A), whereas an evident increase of immunofluorescence was observed along the basal profile of Sertoli cells in 3*g*-mice (Fig 5B). SHBG immunofluorescence can be observed fully expressed also in endocrine Leydig cells; in this case, no difference was detected between control and 3*g*-mice (Fig 5A and 5B). Negative immunohistochemistry control slides resulted completely blank (Fig 5C and 5D).

### RT-qPCR

The effects of exposure to hypergravity in comparison to normogravity condition on the expression of IL1β, FSHR, Androgen Receptor (AR) and, ICSHR were evaluated analysing mRNA expression of these factors. In normogravity, for the several factors analysed, the results showed a low standard deviation of the data around the mean values

**Fig 6:**
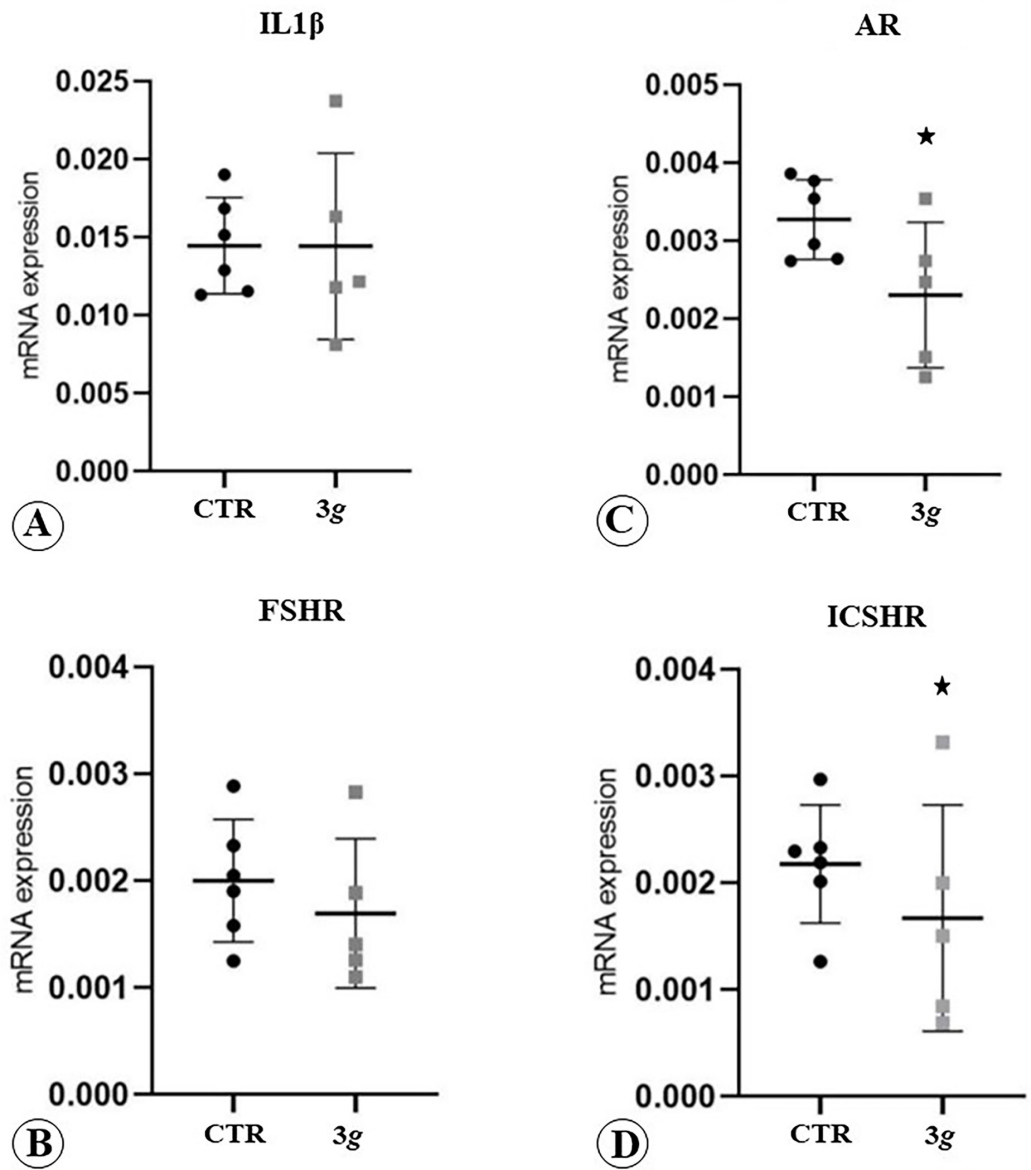
Dot-blot graphs illustrating the mRNA expression in testis seminiferous tubules of several receptors involved in the physiological response of the testis. A: IL1β; B: FSHR; C: AR; D: ICSHR. The star indicates statistical significance.

Exposure to hypergravity induces an increase of the data variability in the IL1β and FSHR mRNA expression, as detected by the larger standard deviation of the values, not accompanied by significative changes of the means values. We hypothesize that these findings may indicate the occurrence of a slight disturbing effect on the IL1β and FSHR-related mRNA expression, which does not reach a significant effect in the experimental condition adopted in the present study (Fig 6A and 6B). Instead an increase of standard deviation, accompanied by statistically significative reduction of mRNA transcript levels was detected for AR and ICSHR (Fig 6C and 6D).

## Discussion

Into the seminiferous tubules, spermatogenic cells are organised to evolve in mature spermatozoa. Many cycles for spermatozoa differentiation occur sequentially along the convoluted seminiferous tubules of the testis, resulting in pulses of sperm release that ensure continued production. Sertoli cells (SC) are constitutive non-spermatogonia cells that have support and trophic functions for the spermatogonia cells of the seminiferous epithelium. SC facilitate transport of mature spermatids towards the lumen of the tubules, provide secretion of androgen binding protein and molecules with endocrine or paracrine action for spermatogenesis. They are responsible to assure the function of the blood-testis barrier. Furthermore, they interact with intertubular endocrine Leydig cells secreting testosterone. Collectively, these tasks make SC elements of paramount importance for the testicular function.

In our study, the first evidence of hypergravity disturbance into testis environment has been obtained by observing the morphology of germinative epithelium. We have observed that hypergravity affects the thickness and the general organization of germinative epithelium of seminiferous tubules, consisting of SC with small nuclei, presence of immature spermatogonia cells with large nuclear profile in the upper layer of germinative epithelium, where instead, only cells with small hyperchromatic nuclei and differentiative cellular profile should be physiologically observed. As a reflex of the regular spermiogenesis process which leads immature spermatogonia cells to differentiate, mainly in the nucleus, into cellular elements presenting nucleus with high condensed haploid genetic content.

Therefore, the maturation and production of spermatozoa display a defective cyto-organization, in fact rarely spermatozoa were observable in the lumen of seminiferous tubules in rats exposed to hypergravity.

The good physiological performance of SC embedded in the spermatogenic layer is widely dependent on cell-cell tight junctions which are made of proteins called occludins and claudin, which interact forming a branching network between the two opposite cells resulting in a tighter seal and mechanical stability. These proteins are linked to elements of cellular cytoskeleton, being able to transduce mechanical cues in intracellular signals. Proteins of tight junctions are involved in maintaining polarity, establishing organ-specific apical domains and participate into SC assisting function during proliferation, differentiation, and migration of spermatogenic cells and realize the blood-testis barrier to control metabolic access to seminiferous tubules environment [16, 17]. We found hypergravity to alter the displaying pattern of claudins and occludin.

We hypothesize that a rearrangement of tight junction among SC has occurred, resulting in an efficiency loss of SC cells in supporting the steps of germ cells differentiation. This alteration in the SC cell-cell contact may be cause of the visible disassembly of the spermatogenic cells disposition with larger spaces between cells and smaller cells closer to larger ones.

In addition to the tight junction complex, which allows stable linkage between adjacent cells, SC layer exhibits several gap junctions, specialized membrane areas which allow cells to communicate each other exchanging small solutes and ions. The gap junction consists of two hemichannels, one for each cell, made up of of sets of six channel proteins called Connexins.

This type of junction offers a pivotal advantage in cell-cell communication because it makes the SC layer working as a functional syncytium, able, of making all SC cells react together, thereby optimizing the physiological behaviour of each tubule.

Following hypergravity treatment the connexin expression was markedly reduced suggesting a probable decrease in cell-cell communication, reflecting a severe trouble in coordinating the cellular response aimed at spermatozoa production. Thus, the occurrence of this type of alterations has an important negative effect on germ cells differentiation culminating in a limited reproduction rate of spermatozoa.

Although we do not have any direct evidence, we can suppose that other epithelial layers or tissues may be subjected to intimate alterations of cell-cell junctions altering the physiological performances under hypergravity conditions.

Blood-brain barrier (BBE) destabilization has been proved in mice following centrifugation treatment at 2*g* showing altered permeability characteristics which represent the main target of the endothelial lining of the BBE to control exchanges between blood and brain parenchyma [18]. Similarly, we expect that in the seminiferous tubules the alteration of cell-cell contact among Sertoli cells could induce an alteration in the blood-testis barrier, compromising the regular surveillance of metabolic traffic between blood and germinative epithelium.

Another important anatomo-physiology compartment in the testis is represented by Leydig cells inside the interstitial stroma representing the glandular compartment producing the greatest quantity of testosterone.

Testosterone biosynthesis is powered by different enzymes the 3β-hydroxysteroid dehydrogenase (3β-HSD) and the 17β-hydroxysteroid dehydrogenase (17β-HSD) being the main ones involved. [19] Our data have shown that hypergravity exposition reduce widely the expression of these two enzymes, suggesting a loss of function of the glandular compartment of the testis.

Testosterone is a powerful hormone involved not only in spermatogenesis physiology, but in upper brain cognitive functions [20], bone metabolism [21], cardiovascular parameters [22], glucose homeostasis [23]. Therefore, a lack in this hormone synthesis can affects multiple physiological pathways. In such case the adrenal gland can compensate for testis function by producing another font of testosterone. At the present we have any evidence concerning a direct effect of hypergravity on other endocrine glands, however, the scientific literature reports following hypergravity exposure, the alteration of the adrenal gland zona reticularis representing the secondary font of production of testosterone [24]. Following these evidence hypergravity seems to decrease widely the testosterone production, and the hypothesis of a wider involvement of hormonal balance may be advanced. Indeed, recent data show that hypergravity conditions could remodel thyrocytes cell membrane by increasing thyrotropin-receptor (TSHR) surface protein [25], but further data concerning the hypergravity effects on hormone receptor expression are still lacking.

In this view, we have analysed the receptor pattern expression for key hormones regulating the physiological function of the testis. We focused on androgen receptor (AR), interstitial cell stimulating hormone receptor (ISCHR), follicle-stimulating hormone receptor (FSHR) and we also detected the expression level of interleukin-1β. AR is a nuclear receptor which reflects the cellular response to testosterone and, in general to androgen hormones, in supporting the normal testis physiology and maintaining the male sexual phenotype. ICSHR is mainly expressed in Leydig cell where it drives testosterone production under hypothalamic-hypophysis axis control. FSHR regulates spermatogenesis and its main function is, upon binding of FSH, to increase the number of Sertoli cells by stimulating their mitotic activity. It is also essential for tight junctions’ formation [26]. IL1β is a pleiotropic cytokine that contributes to the specific immune environment of mammalian testis and in regulating cell differentiation. Our data show only a significant reduction in AR and ICSHR expression, with no changes in FSHR and IL-1β levels. These findings further could confirm the occurrence of a diminished release of testosterone from Leydig cells with the consequent reduction of AR and ICSHR expression.

SC provide key signals to support germ cell survival proceeding through spermatogenesis, and the withdrawal of ICSH and/or testosterone results in the induction of apoptosis at particular stages of germ cell development reducing spermatogenic efficiency [27]. The lack of the effects on FSHR and IL-1β, together with the specific increasing of SHBG expression on SC, could suggest the fall of physiological function of SC toward a quiescent phase, that evolve in a loss of some SC as evidenced by apoptotic signals, leading to an impaired homeostasis of the organ and reduced formation of spermatozoa.

Under the point of view of machine scientific performance, we have obtained a low variation of morphological and molecular data, highlighting the reliability and reproducibility of the data following the use of the Large Diameter Centrifuge machine. Obviously, the size of the cages does not allow to use a large number of animals for single experiments, however the homogeneity of the data obtained is a promising factor for future experiments in which more section of experimental procedures needs to be adopted.

## Conclusions

The present research aimed to investigate morphological and physiological alterations induced by hypergravity in the testis, used as putative model of endocrine gland deregulation.

Our preliminary results show a disassembly in SC lining organization, evidencing that in addition to the morphological alteration of gametogenic epithelium of testis there is also an alteration of surface expressed proteins. This finding may represent, for future exploration on other tissue, an indication oriented towards the analysis of expression pattern of membrane protein, in order to better understand the morphological alteration of the organ or system following altered gravity condition.

Similarly, hypergravity has been shown to reduce hormone level not only by altering the biochemical pathways of hormone production, but also by reducing the expression of receptor membrane proteins. Together these results give strength to the hypothesis that hypergravity, so as microgravity can induce an alteration of cytoskeletal dynamics representing the primary cause of alteration of several cytoplasmic pathways and the expression of membrane protein [2, 28].

Further experiments with longer exposure time, also applied to different animal models can help to visualize the effects of hypergravity on cell and tissue physiology in normal and pathological conditions, unveil opportunity not only to safeguard the physiological homeostasis of astronauts, but also to use hypergravity as a tool to counteract organ pathology [29].
